# Tips and Tricks for Safe Retrieval of Tine-based Leadless Pacemakers

**DOI:** 10.19102/icrm.2021.120606

**Published:** 2021-06-15

**Authors:** Muhammad R. Afzal, Shakeel M. Jamal, Jae H. Son, Jae-Hoon Chung, James Gabriels, Toshimasa Okabe, John D. Hummel, Ralph S. Augostini

**Affiliations:** ^1^Division of Cardiovascular Medicine, The Ohio State University Wexner Medical Center, Columbus, OH, USA; ^2^Department of Internal Medicine, Central Michigan University, College of Medicine, Saginaw, MI, USA; ^3^Department of Internal Medicine, Fairfield Medical Center, Lancaster, OH, USA; ^4^Department of Medicine, Division of Cardiology, New York Presbyterian Hospital-Weill Cornell Medicine, New York, NY, USA

**Keywords:** Elevated threshold, leadless pacemakers, Micra™ transcatheter pacing system, retrieval, snare

## Abstract

As leadless pacing (LP) use is expected to increase, it becomes increasingly essential that operators become familiar with the tools and techniques needed to retrieve an LP successfully. The purpose of this review is to describe a stepwise approach for the successful retrieval of tine-based LP devices, including ways to minimize complications.

## Introduction

The leadless pacemaker (LP) is the most recent technological advancement in the pacemaker industry.^1^ Both the tine-based Micra™ transcatheter pacing system (TPS) (Medtronic, Minneapolis, MN, USA) and the active-fixation Nanostim LP system (Abbott, Chicago, IL, USA) have been used successfully in clinical practice; however, due to premature battery depletion issues, the Nanostim device is no longer commercially available.^1^ The Micra™ TPS, delivered into the right ventricle (RV) using a 23-French (Fr) delivery catheter, has been successfully implanted in more than 15,000 patients worldwide to date (Medtronic Resources, personal communication). The Micra™ Transcatheter Pacing study, the LEADLESS II trial, and multiple real-world investigations have demonstrated the efficacy of LPs in comparison with traditional transvenous devices.^2–4^ Until recently, LPs were implanted in patients with an infrequent need for pacing or in those with permanent atrial fibrillation and complete atrioventricular block.

Multiple studies have reported high rates of procedural success and LP implantation.^3,5–9^ An inadequately placed LP with suboptimal pacing parameters or an embolized LP will need to be retrieved.^10–13^ Although the true incidence of LP dislodgment is unknown, more than 50 cases have already been reported in the literature involving retrieval of the Micra™ TPS.^10,11^ As the use of LPs is expected to grow, it will be increasingly essential that operators become familiar with the tools and techniques needed to retrieve an LP successfully. A considerable worldwide multicenter experience has suggested that multiple approaches can be used to remove an LP.^10^ The purpose of this review is to describe a stepwise approach for the successful retrieval of an LP. Particular emphasis will be given to the retrieval of free-floating devices in the heart or pulmonary vasculature.

## Reasons for retrieval

There are multiple reasons for why retrieval of an LP may be required. One of the most feared complications involving the use of an LP is device dislodgment. A dislodged device can either remain in the RV or embolize to the pulmonary vasculature.^14^ In the event of a dislodgment or embolization, retrieval of the LP is important to minimize the risk of thromboembolic complications. A second indication for device retrieval is the observation of suboptimal pacing parameters during the implant procedure.^11^ Any device with inadequate pacing parameters at the time of implant is expected to have a high risk of device dislodgment and should be immediately retrieved. Recapturing the device during the initial implant procedure minimizes the risk of vascular access complications incurred at the time of a subsequent procedure. Additionally, some of the equipment required for device retrieval is used during the implant procedure.

A third indication for retrieval is in situations where the LP is used as a bridge to a permanent device in patients requiring prolonged antibiotics following the extraction of infected cardiac implantable devices.^12,15,16^ In cases involving transvenous lead extractions in the setting of bacteremia or device-related infections, current practice standards support a waiting period ranging from 72 hours to 14 days between extraction and device reimplantation.^17^ Traditionally, these patients received a temporary transvenous pacemaker or a temporary externalized pacemaker while they completed a course of antibiotics, often in a hospitalized setting. An alternative strategy involves implanting an LP, which allows for early discharge and the completion of antibiotics in the outpatient setting.^17^ After the infection issue has been resolved, patients in need of atrial pacing, cardiac resynchronization, or those who had implantable cardioverter-defibrillators extracted can be reimplanted with permanent transvenous devices. These cases represent the third indication for LP retrieval. Finally, although extremely rare, a fourth indication for removing an LP is in the setting of an infection of the LP itself. Although the incidence of LP infection is exceedingly low due to the lack of a subcutaneous pocket and transvenous leads, the absence of direct contact between the operator and the device, and the parylene coating on the device, limited reports in the literature of retrieval for this indication do exist.^18^

## Techniques for percutaneous retrieval of tine-based leadless pacemakers

The Micra™ TPS is the only LP currently available; thus, the following techniques will focus on percutaneous retrieval of the Micra™ TPS. There are three commonly employed approaches for retrieving this device.

### Vascular access

Each of the percutaneous approaches involves using a Micra™ TPS access sheath, which has 27-Fr and 23-Fr outer and inner diameters.

### Snare selection

Successful retrieval of a Micra™ TPS requires the use of a snare. The snare loop diameter and the snare catheter size are essential factors to consider when choosing between the various techniques for Micra™ retrieval. When snaring through the 23-Fr Micra™ delivery catheter during the initial implant procedure, the largest snare loop diameter that can be used is 5 mm. In contrast, snares with larger loop diameters can be used if the retrieval is performed using a steerable sheath through the 27-Fr (outer diameter) Micra™ delivery sheath.

### Approach 1

**Figure 1:**
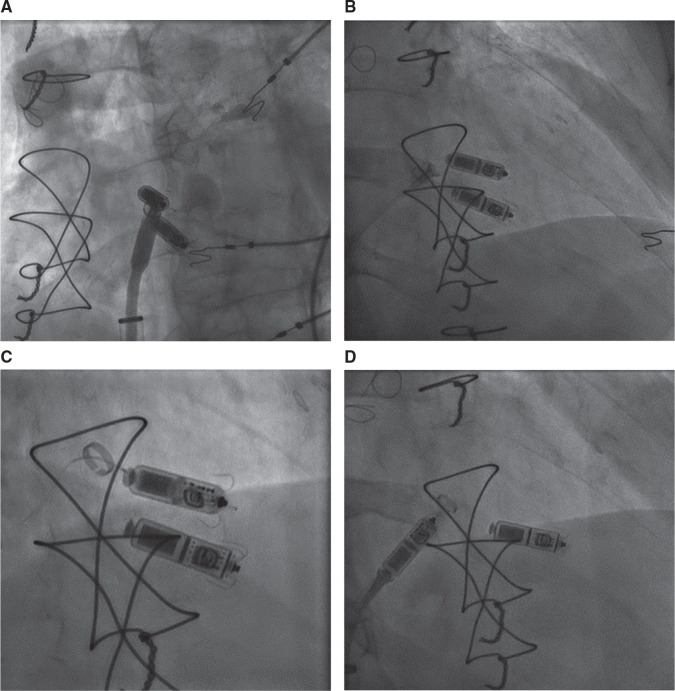
**A–D:** TPS retrieval using a Micra™ delivery catheter and a snare (approach 1). The retrieval was performed after the successful implantation of a new Micra™ TPS in the lower septal location. The Micra™ delivery system cup was advanced toward the proximal end of the Micra™ TPS as visualized in the LAO **(A)** and RAO **(B)** fluoroscopic views. **C:** The snare was engaged on the proximal retrieval feature. **D:** The TPS was withdrawn into the delivery sheath.

If the retrieval is performed during the initial procedure, the Micra™ delivery catheter can be used. This technique involves advancing a 5-mm loop snare through the Micra™ delivery catheter. Once the delivery catheter is in close proximity to the LP, the snare can be advanced over the LP body. To facilitate advancing the snare over the LP, both the right anterior oblique (RAO) and left anterior oblique (LAO) projections should be used **(Figures 1A and 1B)**. Once the snare is around the body of the device, it can be tightened and gradually retracted back to capture the retrieval feature of the LP **(Figure 1C)**. The snare is then cinched and secured by placing hemostatic forceps on the back end. At this point, the LP can be pulled to the edge of the delivery cup and both the device and the delivery catheter assembly are then brought into the outer Micra™ delivery sheath as a unit. This allows the LP to be safely removed from the body.

There are several limitations to using this technique. First, a 7-mm snare loop is the maximal snare size that can be advanced through the Micra™ delivery catheter but can be challenging to engage the LP. Second, the Micra™ TPS delivery catheter has limited steerability, given that the deflection is unidirectional. This limited steerability can hinder adequate coaxial alignment of the catheter with the LP. Despite these limitations, the use of a Micra™ delivery catheter for retrieval has been reported to be successful in an extensive multicenter international experience.^10^

### Approach 2

**Figure 2:**
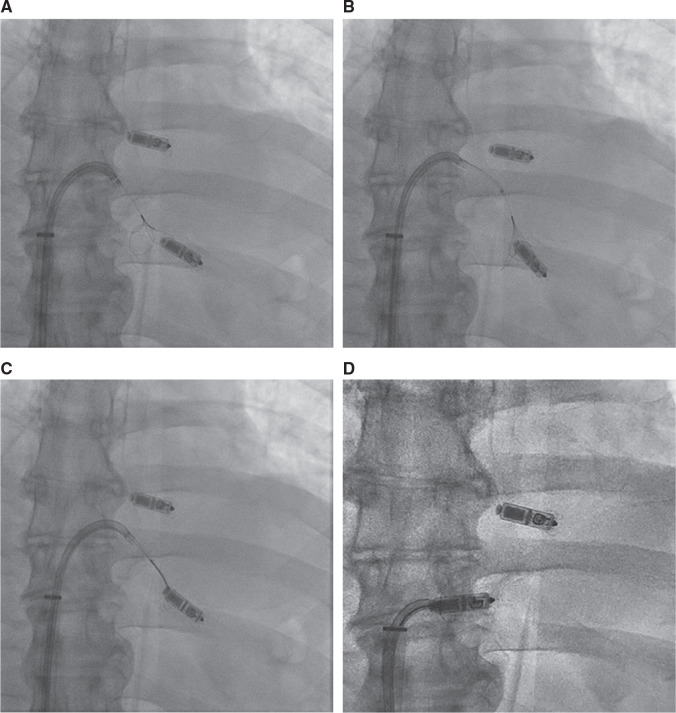
**A–D:** TPS retrieval using a steerable (Agilis NxT™) sheath and snare (approach 2). Retrieval was performed after the successful implantation of a new Micra™ TPS in the septal location, higher than the previous implant. **A:** The steerable sheath with a multiple-loop snare being advanced toward TPS. **B:** The snare was advanced over the body of the TPS. **C:** After confirmation of alignment in multiple views, the snare was tightened around the TPS. **D:** The TPS was pulled to the tip of the steerable sheath and then withdrawn into the delivery sheath.

The most commonly employed approach for retrieving an LP involves the use of an 8.5-Fr steerable sheath (Agilis NxT™; Abbott) inside the 27-Fr Micra™ delivery sheath. A 16-Fr or 14-Fr sheath must be placed inside the 27-Fr sheath prior to inserting the smaller 8.5-Fr steerable sheath to prevent back bleeding from the hemostatic valve. Once the steerable sheath is inside the 27-Fr Micra™ delivery sheath, the system can be advanced into the RV. The 8.5-Fr steerable sheath allows for the use of a larger loop snare (20–30 mm is recommended). The steerable sheath can be brought close to the LP, which facilitates snaring the LP, again using multiple fluoroscopic projections. After the snare has successfully encircled the LP body, it is brought back onto the retrieval feature and tightened. The snare is then withdrawn to the end of the steerable sheath. The LP and the steerable sheath assembly are then brought into the 27-Fr sheath. At this point, the steerable sheath, the LP, and the inner 14- or 16-Fr sheath can be removed from the 27-Fr sheath as a unit. If the LP becomes entrapped in the hemostatic valve of the 27-Fr sheath, the entire apparatus, including the 27-Fr sheath, can be removed from the groin as one unit to prevent dislodgment of the LP in the venous circulation **(Figures 2A–2D)**. Although the use of a steerable sheath is an additional cost, this is a preferred approach for retrieval.

### Approach 3

Retrieval of an LP from the pulmonary arterial vasculature requires the use of additional equipment such as a 5-Fr multipurpose catheter (Merit Medical, South Jordan, UT, USA). Depending on the location of the embolized LP and the size of the steerable sheath, the sheath can be advanced distally into the pulmonary vasculature. The closer the sheath is positioned to the embolized LP, the easier it is to align the sheath with the LP. Depending on the LP location in the pulmonary vascular bed, however, it can be challenging to bring the sheath in close approximation to the device due to the steerable sheath (72-cm working length). If the steerable sheath is not long enough, a multipurpose catheter should be advanced over a Glidewire™ (Terumo Interventional Systems, Somerset, NJ, USA) into the pulmonary artery. After the multipurpose catheter is near the LP, the guidewire can be removed. At this point, a snare can be advanced through the multipurpose catheter and onto the LP. If possible, the snare should be advanced over the body of the LP.

**Figure 3:**
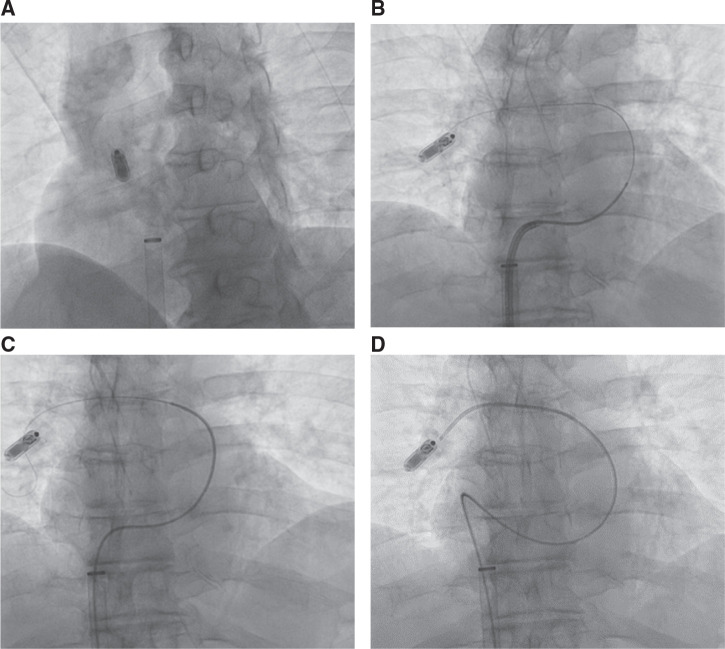
**A–D:** TPS retrieval from the pulmonary artery. **A:** TPS in the right pulmonary artery. **B:** The steerable sheath is advanced into the RV. Due to the limited length of the steerable sheath, a multipurpose catheter was advanced into the RV outflow tract. A single-loop snare was advanced over the TPS; however, the maneuverability was difficult. Despite multiple attempts, the multipurpose catheter could not be advanced into the right pulmonary artery. **C:** A Glidewire™ was advanced into the right pulmonary artery through a multipurpose catheter. **D:** The multipurpose catheter was advanced into the right pulmonary artery and then brought into close proximity to the TPS, using the Glidewire™ as a rail.

**Figure 4:**
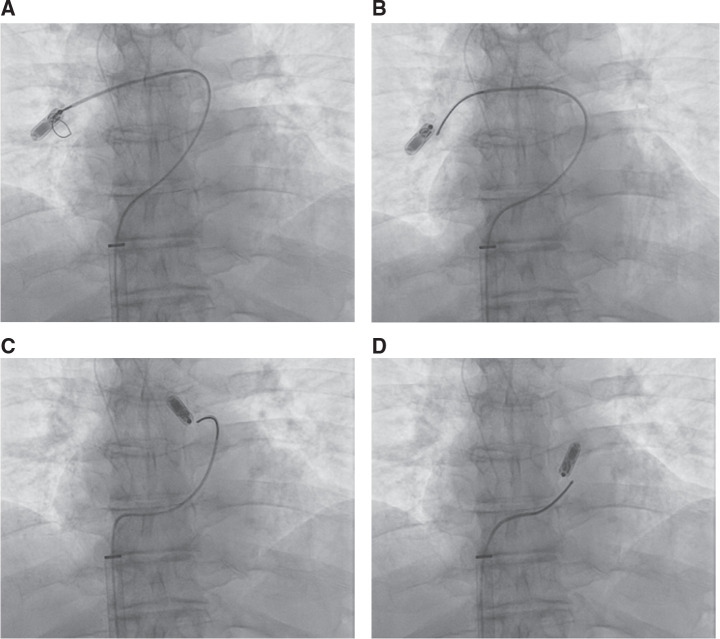
**A–D:** TPS retrieval from the pulmonary artery. A single-loop snare is advanced over the tines **(A)**. The snare was tightened over one of the tines **(B)** and pulled across the pulmonic valve into the RV outflow tract.

**Figure 5:**
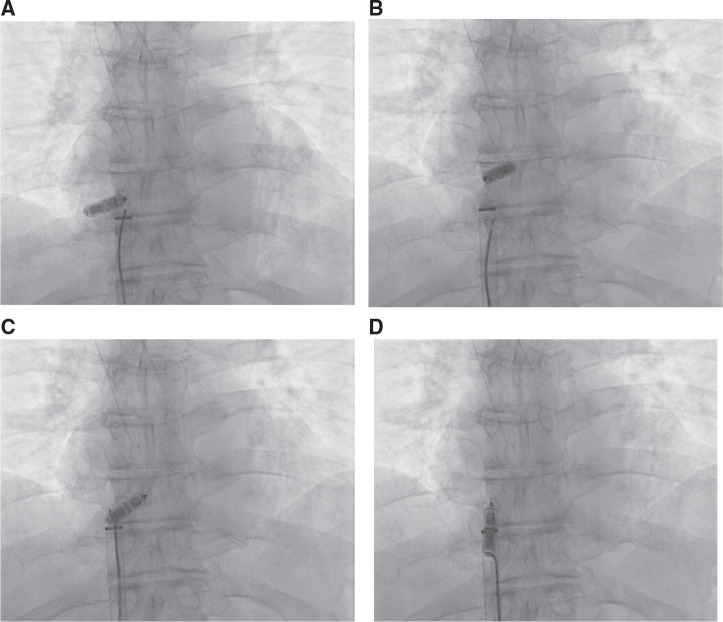
**A–D:** TPS retrieval from the pulmonary artery. During an attempt to pull the TPS into the 27-Fr sheath, the TPS becomes dislodged **(A)**. The single-loop snare is advanced over the body of the TPS. The snare is tightened over the retrieval feature and successfully pulled into the outer sheath.

Occasionally, depending on the embolized LP orientation, the snare may capture one of the LP tines. The snare can be tightened after it has encircled the body of the LP or one of the tines. The multipurpose catheter can then be pulled more proximally into the main pulmonary artery. Two of the authors (M. R. A. and R. S. A.) have successfully used a multipurpose catheter to retrieve an LP from the right pulmonary artery using this technique. In this case, the snare was cinched around one of the tines and was successfully brought into the right atrium. The LP dislodged in the right atrium during an attempt to withdraw it into the 27-Fr sheath. Subsequently, the snare was advanced over the body of the LP and tightened around the retrieval feature, which allowed for a successful retrieval **(Figures 3–5)**. Limitations of this method include potential damage to the pulmonic valve leaflets if the LP is snared in such a fashion that the tines become entangled in the valve apparatus as the device is withdrawn across the valve. While all three of these approaches involve removing the LP through the tricuspid valve, this is the only approach that risks damage to both valves.

### Potential complications during retrieval of an LP

Published experience reports 100% safety during the retrieval procedure.^10,19^ The majority of the patients were able to receive a newer LP with excellent pacing parameters. Long-term follow-up of patients after retrieval was not reported.^10,11^

## Strategies to minimize leadless pacemaker dislodgment

There are multiple steps during LP deployment that can help to minimize the risk of device dislodgment. Most importantly, the deployment site should be confirmed in both RAO and LAO views prior to deploying the device.^17,20^ It is critical to deploy the LP in the heavily trabeculated area of the interventricular septum. The identification of a heavily trabeculated septum is achieved using an iodinated contrast injection in the RAO and LAO views. Ideally, there should be sufficient trabeculations on the superior, anterior, and inferior aspects of the delivery catheter tip. After the LP is deployed in the trabeculated septal area, a good pull-and-hold test should be performed. During this test, the cup of the delivery catheter should come closer to the LP with a visible stretch on at least two of the four tines of the LP.

## Conclusion

The use of LPs is steadily increasing. Although the need for retrieval is infrequent, the skill and familiarity with the required equipment are necessary for any physician who implants these devices. Although multiple approaches can be employed to retrieve an LP, commonly used approaches involve using a steerable sheath and snares of variable loop diameter. Familiarity with the use of snares, in particular, is an essential skillset to successfully and safely retrieve an LP.
